# Duality in nonlinear programming problems under fuzzy environment with exponential membership functions

**DOI:** 10.1186/s13660-018-1812-x

**Published:** 2018-08-22

**Authors:** S. K. Gupta, D. Dangar, I. Ahmad, S. Al-Homidan

**Affiliations:** 1 Department of Mathematics, Indian Institute of Technology, Roorkee, India; 2 Department of Mathematics, Marwari College, Kishanganj, India; 3 Department of Mathematics and Statistics, King Fahd University of Petroleum and Minerals, Dhahran, Saudi Arabia

**Keywords:** 90C26, 90C30, 90C70, 49N15, Fuzzy optimization, Duality results, Exponential membership function, Mangasarian dual

## Abstract

In this paper, we have established appropriate duality relations for a general nonlinear optimization problem under fuzzy environment, taking exponential membership functions and using the aspiration level approach. A numerical example has also been shown to justify the results presented in the paper.

## Introduction

Zadeh in 1965 introduced fuzzy set theory by publishing the first article in this area. He generalized the classical notion of a set and a proposition to accommodate fuzzyness. This has been applied in diverse fields such as machine learning, multi-attribute decision making, supply chain problems, management sciences, etc. Fuzzy control, which directly uses fuzzy rules, is the important application in fuzzy theory. Fuzzy set theory is also applicable in the real life case like controlling smart traffic light. The controller is designed in such a way that it changes the cycle time depending upon the densities of cars behind red and green lights.

The fuzzy set theory provides various logical operators that allow the aggregation of several criteria to just one criterion. These operators can be evaluated with respect to axiomatic requirements, numeric efficiency robustness, degree of compensation among the criteria, and ability to model expert behavior.

Bellman and Zadeh [1] proposed the idea of decision making in fuzzy environment. After the pioneering work on fuzzy linear programming problems (FLPP) in Tanaka et al. [2] and Zimmermann [3], several kinds of (FLPP) along with the different solution methodologies have been discussed in the literature. Many researchers, including Lai and Hwang [4], Shaochang [5], Buckley [6, 7], and Negi [8], have considered the problems where all parameters are fuzzy. Lai and Hwang [4] assumed that the parameters have a triangular possibility distribution. Using multiobjective linear programming methods, they provided an auxiliary model related to it.

Rodder and Zimmermann [9] were the first who studied the duality of (FLPP), considering the economic interpretation of the dual variables. After that, many interesting results regarding the duality of (FLPP) have been investigated by several researchers [10–18]. Zhang et al. [19] investigated the duality theory in fuzzy mathematical programming problems with fuzzy coefficients. Ovchinnikov [20] characterized Zadeh’s extension principle in terms of the duality principle. Introducing the concept of convex fuzzy variables for fuzzy constrained programming, Yang [21] proved a convexity theorem with convex fuzzy parameters and a duality theorem for fuzzy linear constrained programming. Later on, Farhadinia and Kamyad [22] extended the duality theorems for the crisp conic optimization problems to the fuzzy conic programming problems based on the convexity-like concept of fuzzy mappings and the parameterized representation of fuzzy numbers.

The paper is organized as follows. In Sect. 2, we construct a general fuzzy nonlinear programming problem and formulate its Mangasarian type dual. Further, we prove duality theorems using exponential membership functions under convexity assumptions. In the next section, we illustrate a numerical example.

## Definitions and preliminaries

In the crisp sense, a general nonlinear primal-dual pair can be expressed as follows:

*Primal Problem* (*MP*)

$$\begin{aligned}& \mbox{Minimize } f(x) \\& \quad \mbox{s.t. } g_{i}(x)\leq0,\quad i=1,2,\ldots,m, \\& \hphantom{\quad \mbox{s.t. }} x\in X. \end{aligned}$$

*Dual Problem* (*MD*)

$$\begin{aligned}& \mbox{Maximize } L(w,u)=f(w)+ u^{T} g(w) \\& \quad \mbox{s.t. } \nabla f(w)+ u^{T}\nabla g(w)=0, \\& \hphantom{\quad \mbox{s.t. }} u\geq0, \end{aligned}$$

where $X\subseteq R^{n}$, $x, w \in\mathbb{R}^{n}$, $u \in\mathbb {R}^{m}$, $f:R^{n}\rightarrow R$, $g_{i}:R^{n}\rightarrow R$, $i=1,2,\ldots,m$.

Let the aspiration levels corresponding to the objective function of primal (*MP*) and dual (*MD*) be denoted by $z_{0}$ and $w_{0}$, respectively.

Now, the above crisp pair (MP) and (MD) can be described in the fuzzy sense as the following pair (*MP̃*) and (*DD̃*):

*Primal Problem*

$$\begin{aligned}& (\widetilde{\mathit{MP}})\quad \mbox{Find } x\in\mathbb{R}^{n} \mbox{ such that} \\& \hphantom{(\widetilde{\mathit{MP}})\quad}\quad f(x)\lesssim z_{0}, \\& \hphantom{(\widetilde{\mathit{MP}})\quad}\quad g_{i}(x)\lesssim0,\quad i=1,2,\ldots,m, \\& \hphantom{(\widetilde{\mathit{MP}})\quad}\quad x\in X. \end{aligned}$$

*Dual Problem*

$$\begin{aligned}& (\widetilde{\mathit{DD}})\quad \mbox{Find } (w,u)\in\mathbb{R}^{n}{ \times} {\mathbb {R}^{m}} \mbox{ such that} \\& \hphantom{(\widetilde{\mathit{DD}})\quad}\quad L(w,u)=f(w)+ u^{T}g(w) \gtrsim w_{0}, \\& \hphantom{(\widetilde{\mathit{DD}})\quad}\quad \nabla f(w)+ u^{T}\nabla g(w)=0, \\& \hphantom{(\widetilde{\mathit{DD}})\quad}\quad u\geq0, \end{aligned}$$

where “≳” and “≲” are the representations of inequalities “≥” and “≤” in the fuzzy sense, respectively, and have interpretation of “essentially greater than” and “essentially less than” in the sense of Zimmermann.

The exponential membership functions associated with the objective function and the *i*th constraint, $i=1,2,\ldots,m$, are as follows:

$$ \mu_{0}(x) = \textstyle\begin{cases} 1 &\mbox{if } f(x)-z_{0} \leq0, \\ \frac{{e^{-F}-e^{-\alpha}}}{1-e^{-\alpha}}&\mbox{if } 0< f(x)-z_{0}< p_{0}, \\ 0 &\mbox{if } f(x)-z_{0}\geq p_{0} \end{cases} $$

and

$$ \mu_{i}(x) = \textstyle\begin{cases} 1 &\mbox{if } g_{i}(x)\leq0, \\ \frac{{e^{-G_{i}}-e^{-\alpha_{i}}}}{1-e^{-\alpha_{i}}}&\mbox{if } 0< g_{i}(x)< p_{i}, \\ 0&\mbox{if } g_{i}(x)\geq p_{i}, \end{cases} $$

where $F=\alpha((f(x)-z_{0})/p_{0})$, $G_{i}=\alpha_{i}(g_{i}(x)/p_{i})$, and *α*, $\alpha_{i}$, $0<\alpha, \alpha_{i}<\infty$ are fuzzy parameters, also called shape parameters as they measure the degree of vagueness. The constants $p_{0}$, $p_{i}$ ($i=1,2,\ldots,m$) are the allowed change or violations corresponding to the objective function and the constraints of (MP), respectively.

The set of feasible solutions of the fuzzy nonlinear programming problem (*MP̃*) is denoted and defined as follows:

$$\widetilde{D}=\bigl\{ x\in\mathbb{R}^{n}: f(x)\lesssim z_{0}, g_{i}(x)\lesssim0, i=1,2,\ldots,m\bigr\} . $$

It represents the decision space with respect to the fuzzy constraints of (*MP̃*). Its membership function $\mu_{\widetilde{D}} : R \rightarrow[0, 1]$ can be determined from the membership functions of individual fuzzy sets as follows:

$$\mu_{\widetilde{D}}(x)=\min\bigl\{ \mu_{0}\bigl(f(x)\bigr), \mu_{1}\bigl(g_{1}(x)\bigr),\ldots,\mu _{m} \bigl(g_{m}(x)\bigr)\bigr\} . $$

For every $\hat{\mu}_{0}$, $\hat{\mu}_{i}$ lying between 0 and 1, there exist unique $\hat{p}_{0}$, $0<\hat{p}_{0}<p_{0}$ and $\hat{p}_{i}$, $0<\hat{p}_{i}<p_{i}$ such that

$$\mu_{0}(z_{0}+\hat{p}_{0})=\hat{ \mu}_{0}\quad \mbox{and} \quad \mu_{i}(\hat{p}_{i})= \hat{\mu}_{i}. $$

Denote

$$\mathit{FR}(\hat{p}_{0},\hat{p})=\bigl\{ x\in\mathbb{R}^{n}: f(x)\lesssim z_{0}+\hat{p}_{0}, g(x)\lesssim\hat{p}\bigr\} . $$

Let *X* be the universe, whose generic elements are the sets $\mathit{FR}(\hat {p}_{0},\hat{p})$. Then we define a membership function $\mu_{\widetilde {\mathit{FR}}}: X\rightarrow[0, 1]$ by $\mu_{\widetilde{\mathit{FR}}}(\mathit{FR}(\hat{p}_{0},\hat {p}))=\min\{\hat{\mu}_{0},\hat{\mu}_{1},\ldots,\hat{\mu}_{m} \}$. Therefore, the set *D̃* can also be written as

$$\widetilde{D}=\bigcup_{(\hat{p}_{0},\hat{p})}\bigl(\mathit{FR}( \hat{p}_{0},\hat{p}),\mu _{\widetilde{\mathit{FR}}}\bigl(\mathit{FR}( \hat{p}_{0},\hat{p})\bigr)\bigr):\mathit{FR}(\hat{p}_{0}, \hat{p})\in X. $$

Here, *D̃* is a fuzzy set whose elements are the set of points in $R^{n}$ which are generated with the unique aspiration level $z_{0}+\hat{p}_{0}$ and $\hat{p}_{i}$.

Now, following Bellman–Zadeh’s maximization principle and using the fuzzy membership functions defined above, the crisp equivalent of (*MP̃*) can be formulated as follows:

$$\begin{aligned}& \mbox{(PP)} \quad \mbox{Maximize } \xi \\& \hphantom{\mbox{(PP)} \quad}\quad \mbox{s.t. } \xi\leq\frac{{e^{-F}-e^{-\alpha}}}{1-e^{-\alpha}}, \\& \hphantom{\mbox{(PP)} \quad\quad \mbox{s.t. }}\xi\leq\frac{{e^{-G_{i}}-e^{-\alpha_{i}}}}{1-e^{-\alpha_{i}}}\quad (i=1,2,\ldots,m), \\& \hphantom{\mbox{(PP)} \quad\quad \mbox{s.t. }}0 \leq\xi\leq1, \end{aligned}$$

where $F=\alpha((f(x)-z_{0})/p_{0})$ and $G_{i}=\alpha_{i}(g_{i}(x)/p_{i})$, $i=1,2,\ldots,m$.

The above problem can be equivalently expressed as follows:

1 $$\begin{aligned}& \mbox{(PP-1)}\quad \mbox{Maximize } \xi \\& \hphantom{\mbox{(PP-1)}\quad}\quad \mbox{s.t. } p_{0} \log\bigl(\xi+ e^{-\alpha}(1- \xi)\bigr)\leq\alpha\bigl(z_{0}-f(x)\bigr), \end{aligned}$$

2 $$\begin{aligned}& \hphantom{\mbox{(PP-1)}\quad\quad \mbox{s.t. }}p_{i} \log\bigl(\xi+e^{-\alpha_{i}}(1- \xi)\bigr)\leq-\alpha_{i} g_{i}(x)\quad (i=1,2,\ldots,m), \end{aligned}$$

3 $$\begin{aligned}& \hphantom{\mbox{(PP-1)}\quad\quad \mbox{s.t. }}0 \leq\xi\leq1. \end{aligned}$$

Similarly, if the constant $q_{0}$ denotes admissible violations of the objective function of the problem (*DD*), then the crisp equivalent of (*DD̃*) can be obtained as follows:

$$\begin{aligned}& \mbox{(DP)}\quad \mbox{Minimize } -\psi \\& \hphantom{\mbox{(DP)}\quad}\quad \mbox{s.t. } \psi\leq\frac{{e^{-H}-e^{-\beta}}}{1-e^{-\beta}}, \\& \hphantom{\mbox{(DP)}\quad\quad \mbox{s.t. }}\nabla f(w)+ u^{T}\nabla g(w)=0, \\& \hphantom{\mbox{(DP)}\quad\quad \mbox{s.t. }}\psi\in[0,1],\qquad u\geq0, \end{aligned}$$

where $H=\beta((w_{0}-L(w,u))/q_{0})$.

This can be further re-written as

4 $$\begin{aligned}& \mbox{(DP-1)} \quad \mbox{Minimize } -\psi \\& \hphantom{\mbox{(DP-1)} \quad}\quad \mbox{s.t. } q_{0} \log\bigl(\psi+ e^{-\beta}(1- \psi)\bigr)\leq\beta\bigl(L(w,u)-w_{0}\bigr), \end{aligned}$$

5 $$\begin{aligned}& \hphantom{\mbox{(DP-1)} \quad\quad \mbox{s.t. }}\nabla f(w)+ u^{T} \nabla g(w)=0, \\ \end{aligned}$$

6 $$\begin{aligned}& \hphantom{\mbox{(DP-1)} \quad\quad \mbox{s.t. }}\psi\in[0,1],\qquad u\geq0, \end{aligned}$$

where *β* is a shape parameter that measures the degree of vagueness of the objective function of (*DD̃*).

### Theorem 2.1

*For each feasible point of the problem* (*MP̃*) *there exists* *ξ*, $0 \leq\xi\leq1$ *such that* $(x,\xi)$ *satisfies the constraints* (1)*–*(3) *of* (PP-1).

### Proof

Let $x\in\widetilde{D}$. Then there exist some $\hat {p}_{0}$, $0 \leq\hat{p}_{0} \leq p_{0}$, $\hat{p}_{i}$, $0 \leq\hat{p}_{i} \leq p_{i}$, $i=1,2,\ldots,m$, such that $x\in \mathit{FR}(\hat{p}_{0},\hat{p})$ and its membership value is given by $\mu_{\widetilde{\mathit{FR}}}(\mathit{FR}(\hat{p}_{0},\hat {p}))=\min\{\hat{\mu}_{0},\ldots,\hat{\mu}_{m}\}$.

Now, since $x\in \mathit{FR}(\hat{p}_{0},\hat{p})$, therefore we have

7 $$ f(x)\leq z_{0}+\hat{p}_{0},\qquad g(x)\leq \hat{p}. $$

For some $\xi_{0}, \xi_{i}\in[0,1]$, let

$$\begin{aligned}& \hat{p}_{0}=p_{0} \frac{\log(\xi_{0}+ e^{-\alpha}(1-\xi _{0}))}{\alpha}\quad \mbox{and} \\& \hat{p}_{i}=p_{i} \frac{\log(\xi_{i} + e^{-\alpha_{i}}(1-\xi _{i}))}{\alpha_{i}},\quad i=1,2, \ldots,m. \end{aligned}$$

Hence, inequality (7) becomes

$$\begin{aligned}& p_{0} \log\bigl(\xi+ e^{-\alpha}(1-\xi)\bigr)\leq\alpha \bigl(z_{0}-f(x)\bigr), \\& p_{i} \log\bigl(\xi+ e^{-\alpha_{i}}(1-\xi)\bigr)\leq- \alpha_{i}g_{i}(x),\quad i=1,2,\ldots,m, \\& \quad 0 \leq\xi\leq1, \end{aligned}$$

where $\xi=\min(\xi_{0},\xi_{1},\ldots,\xi_{m})$. Therefore, $(x,\xi)$ is feasible for (PP-1). Hence the result. □

### Theorem 2.2

*Suppose* $x_{0}$ *and* $(x_{0},u_{0})$ *are the feasible solutions of* (*MP*) *and* (*MD*), *respectively*. *If the corresponding objective value of* (MP) *fully* (*partially*) *satisfies the goal* $z_{0}$, *then the weak duality theorem between* (*MP̃*) *and* (*DD̃*) *holds* (*partially holds*). *That is*, $z_{0}\geq w_{0}$ ($z_{0}+p_{0}\geq w_{0}-q_{0}$).

### Proof

By the weak duality result between (MP) and (MD), we have

8 $$ f(x_{0})\geq L(x_{0},u_{0}). $$

Since $x_{0}$ is a feasible solution of (MP), therefore we get

$$\begin{aligned} g_{i}(x_{0})\leq0 \quad \mbox{for all } i. \end{aligned}$$

Hence $\mu_{i}(g_{i}(x_{0}))=1$, ∀*i*.

If $f(x_{0})$ fully satisfies the goal $z_{0}$, then $f(x_{0})\leq z_{0}$. Therefore

$$ \mu_{0}\bigl(f(x_{0})\bigr)=1. $$

Also, the membership value of $L(x_{0},u_{0})$,

$$\begin{aligned} \nu\bigl(L(x_{0},u_{0})\bigr)&=\nu\bigl(f(x_{0})+u_{0}^{T}g(x_{0}) \bigr) \\ &=\min\bigl\{ \mu_{0}\bigl(f(x_{0})\bigr), \mu_{1}\bigl(g_{1}(x_{0})\bigr),\ldots, \mu_{m}\bigl(g_{m}(x_{0})\bigr)\bigr\} =1. \end{aligned}$$

So, $L(x_{0},u_{0})\geq w_{0}$.

Combining this with (8) yields

$$ z_{0}\geq f(x_{0})\geq L(x_{0},u_{0}) \geq w_{0}. $$

Now, if $f(x_{0})$ partially satisfies the goal $z_{0}$, then $z_{0}\leq f(x_{0})\leq z_{0}+p_{0}$. Then

$$ \mu_{0}\bigl(f(x_{0})\bigr)\in[0, 1]. $$

That is,

$$\begin{aligned} \nu\bigl(L(x_{0},u_{0})\bigr)&=\nu\bigl(f(x_{0})+u_{0}^{T}g(x_{0}) \bigr) \\ &=\min\bigl\{ \mu_{0}\bigl(f(x_{0})\bigr), \mu_{1}\bigl(g_{1}(x_{0})\bigr),\ldots, \mu_{m}\bigl(g_{m}(x_{0})\bigr)\bigr\} \in[0, 1]. \end{aligned}$$

Hence

$$w_{0}-q_{0}\leq L(x_{0},u_{0})\leq w_{0}. $$

This yields

$$z_{0}+p_{0}\geq f(x_{0})\geq L(x_{0},u_{0})\geq w_{0}-q_{0} \quad \mbox{or}\quad z_{0}+p_{0}\geq w_{0}-q_{0}. $$

This completes the proof. □

### Theorem 2.3

(Modified weak duality)

*Let* $(x,\xi)$ *and* $(w,u,\psi)$ *be feasible solutions for* (PP-1) *and* (DP-1), *respectively*. *Further*, *assume that the functions* *f* *and* *g* *are convex at* *w*. *Then*

$$ \sum_{i=1}^{m} \frac{\log(\xi+ e^{-\alpha_{i}}(1- \xi)) }{\alpha _{i}}{p_{i} u_{i}}\leq f(x)-f(w)-u^{T}g(w). $$

### Proof

Multiplying the constraint (2) of (PP-1) by $u_{i}\geq0$ and further adding all the $`m'$ inequalities, we obtain

9 $$ \sum_{i=1}^{m} \frac{\log(\xi+ e^{-\alpha_{i}}(1- \xi)) }{\alpha _{i}}{p_{i} u_{i}}\leq-u^{T}g(x). $$

By the convexity of *f* and *g* at *w*, we have

10 $$ f(x)-f(w)\geq(x-w)^{T}\nabla f(w) $$

and

11 $$ g(x)-g(w)\geq(x-w)^{T}\nabla g(w). $$

Employing $u\geq0$ in (11) and then adding with inequality (10), we get

12 $$ (x-w)^{T}\bigl(\nabla f(w)+u^{T}\nabla g(w) \bigr)\leq f(x)-f(w)-u^{T}g(w). $$

Finally, using (5) in the addition of (9) and (12), we have

$$ \sum_{i=1}^{m} \frac{\log(\xi+ e^{-\alpha_{i}}(1- \xi)) }{\alpha _{i}}{p_{i} u_{i}}\leq f(x)-f(w)-u^{T}g(w). $$

Hence the result. □

### Theorem 2.4

*Let* $(\bar{x}, \bar{\xi})$ *and* $(\bar{w},\bar{u},\bar{\psi})$ *be feasible solutions for* (PP-1) *and* (DP-1), *respectively*. *Assume that the following conditions hold*:

$$\begin{aligned} (\mathrm{i})&\quad \sum_{i=1}^{m} \frac{\log(\bar{\xi}+ e^{-\alpha_{i}}(1-\bar{\xi})) }{\alpha_{i}}{p_{i} \bar{u}_{i}}= f(\bar{x})-f(\bar{w})- \bar{u}^{T}g(\bar{w}), \\ (\mathrm{ii})&\quad \frac{\log(\bar{\xi}+ e^{-\alpha}(1- \bar{\xi})) }{\alpha }{p_{0}}+\frac{\log(\bar{\psi}+e^{-\beta}(1- \bar{\psi}))}{\beta}q_{0}= \bigl\{ G(\bar{w},\bar{u})-f(\bar{x})\bigr\} +\{z_{0}-w_{0}\}, \\ (\mathrm{iii})&\quad w_{0}-z_{0}\leq0. \end{aligned}$$

*Then* $(\bar{x}, \bar{\xi})$ *and* $(\bar{w},\bar{u},\bar{\psi})$ *are the optimal solutions to* (PP-1) *and* (DP-1), *respectively*.

### Proof

Let $( x,\xi)$ and $(w,u,\psi)$ be feasible solutions for (PP-1) and (DP-1), respectively. Then

$$ \sum_{i=1}^{m} \frac{\log(\xi+ e^{-\alpha_{i}}(1-\xi)) }{\alpha_{i}}{p_{i} u_{i}}+f(w)+u^{T}g(w)-f(x)\leq0. $$

Using hypothesis (i) gives

$$\begin{aligned}& \sum_{i=1}^{m}\frac{\log(\xi+ e^{-\alpha_{i}}(1-\xi)) }{\alpha_{i}}{p_{i} u_{i}}+ f(w)+u^{T}g(w)-f(x) \\& \quad \leq\sum_{i=1}^{m} \frac{\log(\bar{\xi}+ e^{-\alpha_{i}}(1-\bar{\xi})) }{\alpha_{i}}{p_{i} \bar{u}_{i}}-f(\bar{x})+f(\bar{w})+\bar{u}^{T}g(\bar{w})=0. \end{aligned}$$

It follows that $( \bar{x},\bar{\xi}, \bar{w},\bar{u},\bar{\psi})$ is the optimal solution to the following optimization problem whose maximum value is zero:

$$\begin{aligned}& \bigl(\mathit{MP}^{*}\bigr)\quad \mbox{Maximize }\sum _{i=1}^{m} \frac{\log(\xi+ e^{-\alpha_{i}}(1-\xi))}{\alpha_{i}}{p_{i} u_{i}}+ f(w)+u^{T}g(w)-f(x) \\& \hphantom{(\mathit{MP}^{*})\quad}\quad \mbox{s.t. } \frac{\log(\xi+e^{-\alpha}(1- \xi)) }{\alpha}{p_{0}}\leq z_{0}-f(x), \\& \hphantom{(\mathit{MP}^{*})\quad\quad \mbox{s.t. }}\frac{\log(\psi+ e^{-\beta}(1-\psi)) }{\beta}{q_{0}}\leq L(w,u)-w_{0}, \\& \hphantom{(\mathit{MP}^{*})\quad\quad \mbox{s.t. }}\frac{\log(\xi+ e^{-\alpha_{i}}(1- \xi)) }{\alpha_{i}}{p_{i}}\leq-g_{i}(x)\quad ( i=1,2,\ldots,m), \\& \hphantom{(\mathit{MP}^{*})\quad\quad \mbox{s.t. }}\nabla f(w)+ \sum_{i=1}^{m} u_{i}\nabla g_{i}(w)=0, \\& \hphantom{(\mathit{MP}^{*})\quad\quad \mbox{s.t. }}0 \leq\xi\leq1,\qquad 0 \leq\psi\leq1,\qquad u\geq0. \end{aligned}$$

Further, hypotheses (i) and (ii) yield

$$\begin{aligned}& \sum_{i=1}^{m} \frac{\log(\bar{\xi}+ e^{-\alpha_{i}}(1- \bar{\xi})) }{\alpha_{i}}{p_{i} \bar{u}_{i}}+\frac{\log(\bar{\xi}+e^{-\alpha}(1- \bar{\xi})) }{\alpha}{p_{0}} \\& \quad{} +\frac{\log(\bar{\psi}+ e^{-\beta}(1- \bar{\psi}))}{\beta}q_{0}+\{ w_{0}-z_{0} \}=0. \end{aligned}$$

Since $\bar{\xi}, \bar{\psi}\leq1$, therefore each term in the above expression is nonpositive, which sums up to zero. Hence

$$\begin{aligned}& \sum_{i=1}^{m} \frac{\log(\bar{\xi}+e^{-\alpha_{i}}(1- \bar{\xi})) }{\alpha_{i}}{p_{i} \bar{u}_{i}}=0, \\& \frac{\log(\bar{\xi}+ e^{-\alpha}(1- \bar{\xi})) }{\alpha}{p_{0}}=0, \quad \mbox{and} \\& \frac{\log(\bar{\psi}+e^{-\beta}(1- \bar{\psi}))}{\beta}q_{0}=0. \end{aligned}$$

Since

$$ \frac{\log(\xi+ e^{-\alpha}(1- \xi)) }{\alpha}{p_{0}}\leq0= \frac{\log(\bar{\xi}+ e^{-\alpha}(1- \bar{\xi})) }{\alpha}{p_{0}}, $$

therefore

$$ \log\bigl(\xi+ e^{-\alpha}(1- \xi)\bigr)\leq \log\bigl(\bar{\xi}+ e^{-\alpha}(1- \bar{\xi})\bigr), $$

which implies $\xi\leq\bar{\xi}$. Similarly, we obtain $-\psi\geq-\bar{\psi}$. This proves the result. □

## Numerical illustration

Consider the following primal-dual pair:

$$\begin{aligned}& \mbox{(P)}\quad \mbox{Minimize } h(x,y)=x+2x^{2}+y^{2} \\& \hphantom{\mbox{(P)}\quad}\quad \mbox{s.t. } 2x+2y\geq1. \end{aligned}$$

and

$$\begin{aligned}& \mbox{(D)} \quad \mbox{Maximize } H(w,z,u)=w+2w^{2}+z^{2}+u(1-2w-2z) \\& \hphantom{\mbox{(D)} \quad}\quad \mbox{s.t. } 4w-2u+1=0, \\& \hphantom{\mbox{(D)} \quad\quad \mbox{s.t. }}u-z=0, \\& \hphantom{\mbox{(D)} \quad\quad \mbox{s.t. }}u\geq0. \end{aligned}$$

Taking an exponential membership function in the primal problem (P) and the dual problem (D), the corresponding problems (PP-1) and (DP-1) become:

$$\begin{aligned}& \mbox{(FP)}\quad \mbox{Maximize } \xi \\& \hphantom{\mbox{(FP)}\quad}\quad \mbox{s.t. } \log\bigl(\xi+e^{-2}(1- \xi)\bigr) \leq\bigl(1-x-2x^{2}-y^{2}\bigr), \\& \hphantom{\mbox{(FP)}\quad\quad \mbox{s.t. }}2 \log\bigl(\xi+ e^{-1}(1- \xi)\bigr)\leq(2x+2y-1), \\& \hphantom{\mbox{(FP)}\quad\quad \mbox{s.t. }}\xi\in[0,1], \end{aligned}$$

and

$$\begin{aligned}& \mbox{(FD)}\quad \mbox{Minimize } -\psi \\& \hphantom{\mbox{(FD)}\quad }\quad \mbox{s.t. } \log\bigl(\psi+e^{-2}(1- \psi)\bigr) \leq2\bigl(w+2w^{2}+z^{2}+u-2uw-2uz-1\bigr), \\& \hphantom{\mbox{(FD)}\quad \quad \mbox{s.t. }}4w-2u+1=0, \\& \hphantom{\mbox{(FD)}\quad \quad \mbox{s.t. }}u-z=0, \\& \hphantom{\mbox{(FD)}\quad \quad \mbox{s.t. }}\psi\in[0,1],\qquad u\geq0, \end{aligned}$$

where $p_{0}=p_{1}=2$, $z_{0}=1$, $\alpha=2$, $\alpha_{1}=1$ and $q_{0}=1$, $\beta=2, w_{0}=1$. Using a software MAPLE 12, the optimal solutions of (FP) and (FD) are $x^{*}=0.3190 $, $y^{*}=0.6910$, $\xi^{*}=1 $ and $\psi^{*}= 0.1016$, $u^{*}= z^{*}=0.50$, $w^{*}=0$, respectively. Since all the assumptions of Theorems 2.3 and 2.4 are satisfied for the above problems, hence at these points the results of the theorems can easily be verified.
